# Rigid side-hole cap-assisted guidewire fragmentation of giant gastric bezoars

**DOI:** 10.1055/a-2891-6057

**Published:** 2026-06-25

**Authors:** Jiaju Li, Chenghong Li, Yachun Dai, Hong Zhang, Chunye Hu, Xiao Hu

**Affiliations:** 1 Department of Gastroenterology and HepatologyPeople’s Hospital of Weining Yi, Hui and Miao Autonomous CountyWeining Yi, Hui and Miao Autonomous CountyGuizhouChina; 2 Department of Gastroenterology and Hepatology 89669 Sichuan Provincial People’s Hospital, School of Medicine, University of Electronic Science and Technology of ChinaChengduSichuanChina

Fragmentation of giant hard gastric bezoars remains challenging when conventional snare extraction fails. Failure of a large snare may reflect giant size preventing stable capture, slippage during tightening, and insufficient cutting force from the plastic snare sheath against a hard bezoar. Previously reported guidewire-assisted methods often require dedicated accessories, accessory modification, 1​ ​2​ or double-channel endoscopes. 3​ ​4​ We report a rescue technique using a rigid transparent side-hole cap and a standard guidewire.

**Fig. 1:**
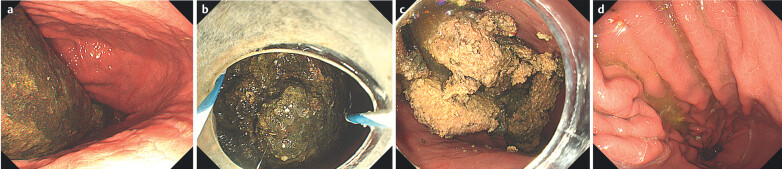
Endoscopic fragmentation and clearance of giant gastric bezoars. ( **a** ) Two giant hard phytobezoars in the gastric body. ( **b** ) The adjustable guidewire loop captures the bezoar. ( **c** ) Traction against the rigid cap edge creates a guillotine-like fragmentation effect. ( **d** ) Complete clearance is confirmed at a 1-week follow-up.

A 62-year-old woman presented with epigastric pain and vomiting for 3 days after heavy persimmon consumption. Esophagogastroduodenoscopy revealed two giant hard phytobezoars in the gastric body, measuring approximately 4×6cm and 6×10 cm ( Video 1 ; Fig. 1a ). Initial extraction with a large 6×3 cm snare failed.

**Video 1** Rigid side-hole cap-assisted guidewire fragmentation of giant gastric bezoars after failed conventional snare extraction.


**Fig. 2:**
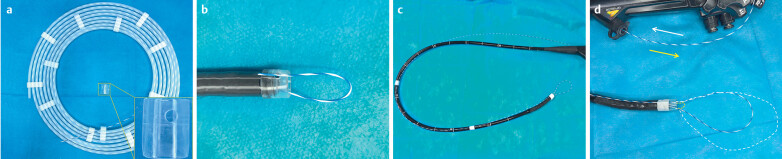
Schematic illustration of the rigid side-hole cap-assisted guidewire technique. ( **a** ) The setup includes a rigid transparent side-hole cap and a standard guidewire. ( **b** ) A 0.025-inch guidewire is advanced through the working channel and exits from the distal end of the endoscope. ( **c** ) The guidewire is folded back, passed from inside the cap through the side hole, aligned parallel to the shaft, and fixed with tape. ( **d** ) By advancing or withdrawing the intrachannel end of the guidewire, an adjustable large loop is created according to the bezoar size.

A single-channel gastroscope was first fitted with a rigid transparent side-hole cap. For assembly, a 0.025-inch guidewire was advanced through the working channel and brought out from the distal end of the endoscope. The wire was then folded back to form a loop, passed from inside the cap through the side hole, aligned parallel to the endoscope shaft, and fixed with tape. By advancing or withdrawing the intrachannel end of the guidewire, a large adjustable loop could be created according to the bezoar size. During fragmentation, one wire limb remained within the channel and the other outside; however, the thin guidewire occupied minimal channel space and did not materially impair suction or accessory passage. Traction against the rigid cap edge generated a guillotine-like cutting effect, allowing repeated fragmentation ( Fig. 1b, c ). The total procedure time was 35 minutes, with no adverse events. Follow-up endoscopy 1 week later confirmed complete clearance ( Fig. 1d ). The assembly of the rigid side-hole cap-assisted guidewire system is shown in Fig. 2 .

This rigid side-hole cap-assisted technique may represent a practical rescue option without dedicated accessories or double-channel endoscopes.

Endoscopy_UCTN_Code_TTT_1AO_2AN
